# Assessing AlphaFold 3 for Per- and Polyfluoroalkyl Substances Docking in Protein Structures

**DOI:** 10.1021/acs.est.5c03917

**Published:** 2025-08-26

**Authors:** Xiping Gong, Hualu Zhou, Qingguo Huang

**Affiliations:** † Department of Crop and Soil Sciences, College of Agricultural and Environmental Sciences, 1355 University of Georgia, Griffin, Georgia 30223, United States; ‡ Department of Food Science and Technology, College of Agricultural and Environmental Sciences, 1355 University of Georgia, Griffin, Georgia 30223, United States

**Keywords:** molecular docking, autoDock vina, protein-PFAS interactions, in-silico binding prediction, PFAS types

## Abstract

Per- and polyfluoroalkyl substances (PFAS) are persistent environmental pollutants that may pose health risks due to strong protein interactions. While AlphaFold 3 (AF3) was recently introduced for protein–ligand modeling with high claimed accuracy, its reliability for docking PFAS remains unclear. This study evaluates AF3′s performance in predicting protein–PFAS interactions using a curated data set from the Protein Data Bank, divided into a “Before Set” (seen during AF3 training) and an “After Set” (unseen). AF3 accurately predicts protein structures and pockets but shows reduced performance in pocket-aligned ligand predictions, achieving ∼74.5% success in “Before Set” but only ∼55.8% in “After Set”, indicative of possible overfitting. We further assess the effects of PFAS type on docking outcomes. Although AF3 accurately predicts binding pockets, it favors poses where the headgroup of environment-relevant PFAS interacts with polar or positively charged residues. This is different from another native binding mode in several cases, where the hydrophobic tail is inserted in the protein, but the headgroup is exposed to the solvent. Notably, a hybrid approach combining AF3 and Vina, especially considering multiple top-ranked poses, can improve prediction accuracy. These findings support the complementary use of AF3 and Vina for accurately modeling protein–PFAS interactions.

## Graphical abstract

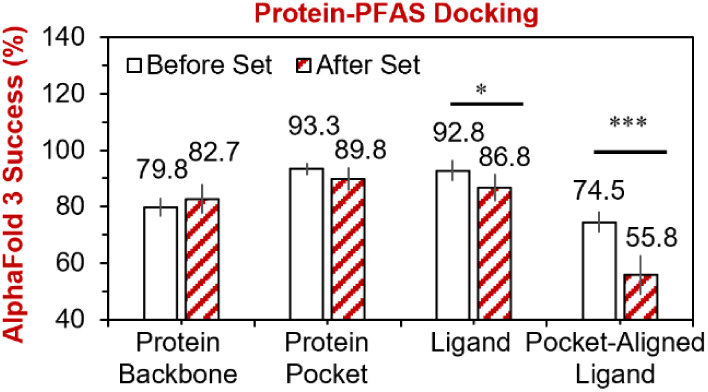

## 1 Introduction

Per- and polyfluoroalkyl substances (PFAS) are a large class of synthetic fluorinated compounds, with over ten thousand known structures.^1^ They have been used in industrial and consumer applications due to their chemical stability, hydrophobicity, and resistance to heat and degradation.^2,3^ These properties have led to their widespread use in products such as firefighting foam, nonstick cookware, waterproof fabrics, and food packaging, but the same stability also contributes to their persistence in the environment, leading to widespread contamination of water, soil, and air.^4,5^ PFAS exposure has been linked to various adverse health effects, including endocrine disruption, immune system suppression, liver toxicity, and an increased risk of certain cancers.^6,7^ Additionally, PFAS bioaccumulate in humans and wildlife, further compounding their environmental and public health risks.^8–11^

Given their persistence and potential toxicity, understanding how PFAS interact with biological systems, particularly proteins, is crucial for assessing the biological impact of these persistent contaminants. Once released into the environment, PFAS can accumulate in living organisms, where they have been detected in blood, liver, and other tissues.^12^ Many PFAS compounds structurally resemble fatty acids and other biological ligands, allowing them to bind to key proteins such as serum albumin, enzymes, and hormone receptors, which could disrupt normal protein function, altering metabolic processes, immune responses, and endocrine signaling.^13–15^ Additionally, protein binding plays a critical role in PFAS transport, distribution, and elimination in biological systems, influencing their toxicity and long-term effects.^16^ Therefore, studying protein-PFAS interactions is essential for predicting their biological behavior, identifying potential health risks, and developing effective strategies for risk assessment and remediation.

Traditional experimental techniques provide valuable insights but are often time-consuming, costly, and limited in their ability to explore a large number of PFAS compounds and protein targets. Using in-silico methods has become an effective strategy to study protein-PFAS interactions due to their ability to rapidly screen and predict binding behaviors at the molecular level.^17,18^ For example, both molecular docking and molecular dynamics simulations allow researchers to analyze binding affinities, interaction mechanisms, and structural conformations of PFAS within protein binding sites in a high-throughput manner.^19,20^ These methods can also provide atomic-level insights into key molecular forces, such as hydrophobic interactions and electrostatic effects, which drive PFAS binding. Additionally, in-silico tools can be integrated with experimental studies to refine predictions and prioritize targets for further investigation.^21^ Particularly, molecular docking enables high-throughput screening of numerous PFAS compounds against a wide range of protein targets.^20,22^ By modeling the three-dimensional structures of proteins and PFAS molecules, docking algorithms predict the most favorable binding poses based on complementary shape, electrostatic interactions, and hydrophobic forces.^23^ Additionally, molecular docking serves as a foundational step for more advanced simulations, such as molecular dynamics, helping to refine predictions and improve accuracy. Given the structural diversity of PFAS and their widespread biological presence, molecular docking is an essential tool for elucidating their potential interactions with proteins.

AlphaFold 3 (AF3) has recently emerged as a cutting-edge tool for modeling protein–ligand interactions with high accuracy, demonstrating promising results in predicting binding sites.^24^ Unlike traditional docking methods that rely on predefined scoring functions, AF3 integrates deep learning to capture complex protein–ligand interactions with greater sophistication. However, while AF3 has been validated for some biological systems, its capability in docking PFAS into protein structures remains largely untested. A detailed assessment of AF3′s performance in protein-PFAS docking is necessary to determine whether it can reliably capture these interactions or if it suffers from challenges in predicting the protein-PFAS binding.

In this study, we assess the capability of AF3 in docking PFAS within protein structures and compare its performance with the traditional molecular docking tool, AutoDock Vina. To evaluate this, we compiled a protein-PFAS data set from the Protein Data Bank (PDB), categorizing structures into two sets: “Before Set” (data included in AF3′s training) and “After Set” (data unseen by AF3). We then performed docking analyses using both AF3 and Vina to compare their predictive performance. Additionally, we explored a hybrid strategy that combines AF3 for binding pocket identification with Vina for interaction modeling. Through these comparative analyses, this study aims to determine whether AF3 can serve as a reliable tool for PFAS docking and how its prediction accuracy can be improved.

## 2 Methods

### 2.1 Protein-PFAS Data Set Collected from PDB

To construct the data set for docking calculations, we first identified relevant protein-PFAS complexes using the OECD (2021) definition of PFAS.^25^ A total of 2805 PDB entries were initially identified, and 2765 corresponding PDB files were successfully downloaded (as of 2/14/2025). To ensure consistency and simplify the study, we restricted our selection to systems containing one protein and one PFAS molecule, resulting in 1086 protein-PFAS complexes for further analysis. During the docking calculations, some of these complexes failed in either AF3 or AutoDock Vina due to issues such as docking failures or errors in structure preparation and prediction. Complexes containing nonstandard amino acids were also excluded, and only proteins with more than 50 residues were retained to focus on larger macromolecular complexes. As a result, the final data set was refined to 722 protein-PFAS systems, which successfully completed docking simulations using both methods.

To investigate the impact of training data set on AF3 docking performance, we further divided the data set into two subsets: “Before Set” and “After Set”. The classification was based on the release date of the PDB structure, ensuring that only proteins deposited before 09/30/2021 were included in the “Before Set”, while the structures released after that date were assigned to the “After Set”. This assignment enabled a systematic evaluation of AF3′s generalization ability by comparing its performance on training data versus newly deposited entries. A general workflow was provided in the Supporting Information, and the finalized data sets were also used to evaluate the docking performance of AF3 and Vina (Figure S1).

### 2.2 Computational Details of Both AF3 and AutoDock Vina Docking

Molecular docking was performed on the protein-PFAS data set using two docking methods: AF3 and AutoDock Vina. To ensure structural integrity and consistency in molecular docking analysis, PDB file preprocessing was performed using the PDBFixer Python package, which was employed to restore missing heavy atom coordinates in some protein structures.^26^ For systems containing additional molecules, such as water and ligands, these molecules were removed to simplify the docking process. This step was essential for accurate molecular docking and RMSD calculations, as incomplete structural data could introduce errors in docking predictions.

For the AF3 calculations, we used the protein sequence and ligand Chemical Component Dictionary (CCD) code from the native PDB structures as input. However, the CCD codes do not always accurately represent the deprotonated (negatively charged) states of some ligands. To investigate this potential impact, we selected seven extra environment-relevant PDB structures (4E99, 7AAI, 7FD7, 7FEU, 7FEK, 7Z57, and 8U57), which have the negatively charged PFAS at pH 7.4, and generated ligand inputs using SMILES strings that explicitly define the negatively charged form. These modified ligands were then used in parallel AF3 predictions to evaluate the effect of ligand charge state on docking outcomes. For systems containing multiple PFAS ligands, one PFAS molecule was used for docking to simplify the process for both AF3 and Vina, unless otherwise specified in the text. For each system, a single seed was used to generate five models. AF3 docking calculations were executed on a computational cluster equipped with 32 CPUs (Intel­(R) Xeon­(R) Gold 6230 CPU @2.10 GHz) and one NVIDIA A100 GPU, enabling efficient structure generation and docking predictions.

In contrast, three Vina-based strategies are considered: Vina uses the best AF3-predicted pose as input; Vina-1 is an improved version that replaces the AF3-predicted protein structure with the native protein structure while retaining the ligand conformation from the AF3-predicted pose; Vina-2 uses the native complex structure as input, including both the native protein and the native ligand structures. The binding pocket was identified by selecting protein backbone heavy atoms within 1 nm of the geometric center of ligand heavy atoms. Vina docking calculations were performed with an exhaustiveness parameter set to 32, and five docking models were generated per system for subsequent analysis. To prepare input structures for Vina, we used the “mk_prepare_receptor.py” and “mk_prepare_ligand.py” scripts to convert protein and ligand structures into the pdbqt format required for docking.^27^ For all Vina docking calculations, we assume that the input protein structure is fixed. To minimize potential atomic overlaps, we performed geometry optimization of the protein structure using the AMBER14SB force field in GROMACS, and the maximum energy threshold was reduced below 100 kJ/mol/nm^2^.^28,29^ Additionally, we utilized Open Babel for file format conversions, including converting PDB files to SDF files and other necessary transformations to ensure compatibility across docking pipelines.^30^ These preprocessing steps were critical to maintaining consistency and ensuring accurate docking calculations. We used the command “obabel -p 7.4” to assign the protonation state of each ligand at physiological pH. The sum of the calculated partial atomic charges was then used to determine the net charge of each PFAS molecule.

### 2.3 Success Rate, Visualization, Data Classification, and Statistical Analysis

For docking evaluation, the success rate is defined as the percentage of samples with a heavy-atom RMSD ≤ 0.2 nm. The MDTraj Python package was used to compute the aligned RMSD values, ensuring that the ligand was properly superimposed for accurate comparisons.^31^ In addition, an alternative direct method was applied to compute pocket-aligned ligand RMSD values without alignment, to assess ligand placement deviations. The Visual Molecular Dynamics (VMD) was used to render and visualize the docked protein–ligand complexes.^32^

All data processing and analysis were performed using Bash and Python scripts, which are publicly available for reproducibility on a GitHub repository.^33^ To categorize PFAS types, we applied multiple classification criteria, including the OECD (2021) definition (which includes PFAS compounds containing −CF_2_– or −CF_3_ groups), the U.S. EPA OPPT definition (which specifically identifies −CF–CF– motifs), and a separate classification for PFAS with an aromatic fluorine motif (−Ph–F).^34^ These classifications enabled a systematic evaluation of docking performance across different PFAS chemical structures, allowing for a deeper understanding of how molecular features influence docking accuracy. We also categorized the PFAS into three different groups (negative, neutral, and positive) to investigate the impact of the net charge of PFAS on the AF3 predictive accuracy.

Error bars represent 95% confidence intervals, calculated from 10,000 bootstrap resamples. To evaluate success rate, each sample was assigned a value of 1 if the RMSD was ≤0.2 nm, and 0 otherwise. Statistical significance between two samples (*p < 0.05, **p < 0.01, ***p < 0.001) was assessed using Welch’s *t*-test, performed in Python with the scipy.stats.ttest_ind function (equal_var = False) to account for unequal variances. It is noted that Vina results can be nondeterministic. To assess this, we performed two identical Vina runs and found no significant difference in the success rates.

## 3 Results and Discussion

### 3.1 Predictive Accuracy Evaluation of AF3

#### 3.1.1 Success Rate of AF3 in Predicting Protein Structures and Protein-PFAS Interactions

To evaluate the predictive performance of AF3 in modeling protein–PFAS complexes, we compared the success rates of structural predictions on two data sets: the “Before Set” and “After Set.” We assessed the predictions using four structural alignment references: protein backbone, protein pocket, ligand, and pocket-aligned ligand (Figure 1). Both the top-ranked pose (Best Pose) and the top five predicted poses (Top-5) were examined to evaluate prediction consistency and robustness.

**Figure 1:**
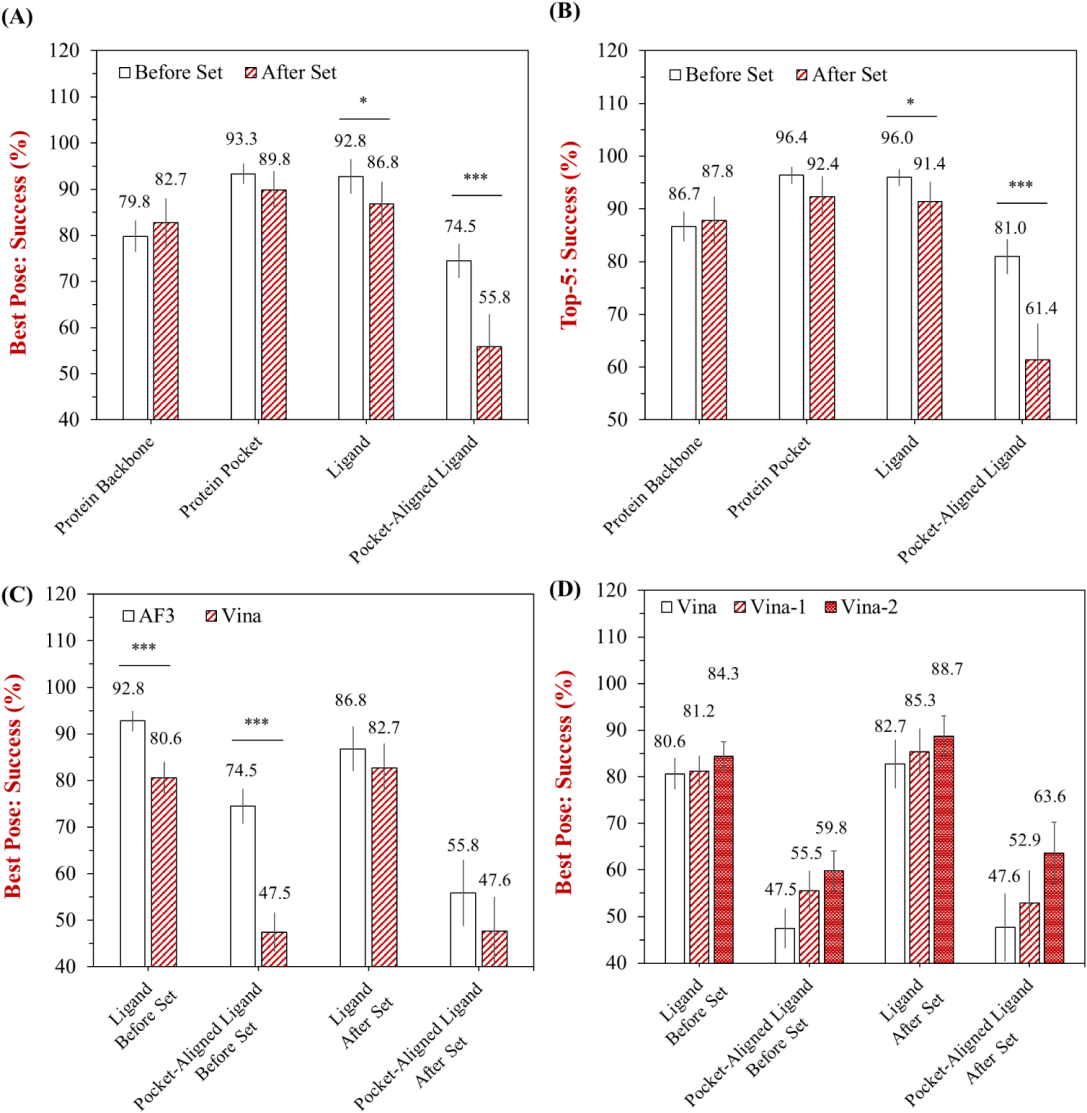
(A,B) Success rates (%) of AF3 on the “Before Set” and “After Set”, and (C,D) comparison of AF3 with different AutoDock Vina docking strategies in terms of four different structural alignment references. Success rate is defined as the percentage of samples with a heavy-atom RMSD ≤ 0.2 nm, in terms of four different structural alignment references: protein backbone, protein pocket, ligand, and pocket-aligned ligand. (A) Percentage of successful predictions for the top-ranked pose (“Best Pose: Success (%)”). (B) Percentage of successful predictions among the top-5 ranked poses (“Top-5: Success (%)”). (C) Comparison between AF3 and Vina on the “Before Set” and “After Set”. (D) Further comparison among three Vina-based strategies across the same conditions. Average success rates are shown above each bar, with error bars representing the 95% confidence intervals. Statistical significance between the two data sets was evaluated using Welch’s *t*-test (*p < 0.05, **p < 0.01, and ***p < 0.001).

In the Best Pose evaluation, AF3 showed strong performance in predicting protein structures, with success rates of 79.8% and 82.7% for protein backbone prediction in both “Before Set” and “After set”, respectively, which is consistent with previously reported results.^24^ Predictions based on the protein pocket and ligand alignments achieved even higher success rates in the “Before Set”, reaching 93.3% and 92.8%, respectively. However, when assessed using a more stringent pocket-aligned ligand reference, the success rate dropped to 74.5%, which was similarly observed in previous results.^24^ This suggests that while AF3 performs reliably in predicting protein structures, its accuracy can be reduced when modeling protein–PFAS complexes. This limitation may be attributed to the scarcity of protein–PFAS complexes in the training data, as well as the vast and diverse interaction space between PFAS molecules and protein residues. Notably, a more pronounced decline was observed in the “After set”, particularly for ligand and pocket-aligned ligand alignments. The pocket-aligned ligand success rate dropped to 55.8%, representing a significant decrease (*p* < 0.001), suggesting a potential overfitting issue in AF3′s predictions (Figure 1A). One likely explanation is that AF3’s performance is partially influenced by memorization or overfitting to familiar protein folds and ligand-binding patterns present in its training data. As a result, when encountering less-represented or structurally distinct PFAS compounds, AF3 may fail to fully capture the nuanced interactions necessary for accurate ligand placement.

As expected, performance improved in the Top-5 evaluation due to the consideration of multiple candidate poses. For instance, success rates for protein pocket and ligand alignments in the “Before Set” reached 96.4% and 96.0%, respectively, compared to 93.3% and 92.8% in the Best Pose evaluation (Figure 1B). These improvements reflect AF3′s excellent capability to generate at least one accurate pose among the top-5 ranked predictions. However, the overall trend of reduced performance in the “After Set” remained consistent. Signs of overfitting were again observed, particularly in the pocket-aligned ligand evaluation, where the success rate declined from 81.0% in the “Before Set” to 61.4% in the “After Set”. These findings highlight the direction for further optimization of AF3, especially for applications demanding high precision in modeling protein–PFAS interactions.

#### 3.1.2 Comparison with AutoDock Vina in Protein–PFAS Docking

To further assess AF3′s performance in protein–PFAS docking, we compared its predictions with those generated using AutoDock Vina under different docking strategies (Figure 1C,D). Since AF3 operates in a blind docking manner, without prior knowledge of protein-PFAS structures and the binding site, it is reasonable to use AF3-predicted protein–PFAS complexes as input structures for Vina, ensuring a fair basis for comparison. In this initial evaluation, AF3 consistently outperformed Vina across both the “Before Set” and “After Set” under all structural alignment references. For example, under the ligand-based alignment criterion, AF3 achieved success rates of 92.8% and 86.8% for the “Before Set” and “After Set”, respectively, while Vina reached lower success rates of 80.6% and 82.7% (Figure 1C). Similarly, under a pocket-aligned ligand criterion, AF3 achieved 74.5% and 55.8% success rates, whereas Vina lagged behind with 47.5% and 47.6%, respectively (Figure 1C). Although conventional docking methods like Vina performed less accurately when applied to AF3-generated structures, an interesting observation is that Vina-refined predictions did not exhibit the same overfitting behaviors, showing relatively consistent performance across both “Before Set” and “After Set”.

It is noted that many factors can affect the performance of Vina in docking PFAS in proteins, such as the input structures of both protein and ligand, as well as the parameters of pocket to dock. To better understand the influence of input structures on Vina’s performance, we introduced two additional docking strategies: Vina-1 and Vina-2. Unlike the original Vina approach, both Vina-1 and Vina-2 use the native protein structure as input. The key distinction between them lies in the ligand input: Vina-1 uses the ligand pose predicted by AF3, while Vina-2 uses the ligand conformation derived from the native PFAS–protein complex. Compared to the original Vina setup, both Vina-1 and Vina-2 showed improvements, particularly in the pocket-aligned ligand alignment. For example, in the “Before Set”, the success rate improved from 47.5% (Vina) to 55.5% (Vina-1) and further to 59.8% (Vina-2), approaching AF3′s performance (Figure 1D). A similar trend was observed in the “After Set”, with Vina-2 yielding the highest success rate among the Vina variants. These results suggest that Vina’s performance can be enhanced by incorporating additional structural information, especially when native or high-quality protein and ligand structures are used. Although the ligand was allowed to be flexible during docking, we observed that the outcomes of both Vina-1 and Vina-2 still varied. This indicates that the starting pose of the ligand plays an important role in the final docking result. The difference may be attributed to the stochastic nature of Vina’s search algorithm, which is influenced by the initial ligand conformation. However, compared to AF3′s results, the refinements introduced by Vina still fall short of significantly improving its overall ability to predict protein–PFAS interactions.

#### 3.1.3 An Overfitting Problem Observed in AF3

To further assess whether AF3′s ligand docking performance exhibits potential overfitting, we further compared the RMSD distributions across two sets (Figure S2). The results show that AF3 performs better on the “Before Set” than on the “After Set”. Specifically, the RMSD distribution for AF3 in the “Before Set” (dashed black line) is more concentrated in the lower RMSD range, indicating higher docking accuracy. However, in the “After Set” (solid black line), the RMSD values shift toward higher deviations, signifying reduced predictive reliability. Vina-2, on the other hand, maintains a relatively stable distribution across both sets, as shown by the red dashed and solid lines, reinforcing its consistency in docking performance. These results indicate that while AF3 can achieve high accuracy under certain conditions, its performance is less reliable when applied to new protein-PFAS complexes.

Despite its tendency to overfit, AF3 remains a valuable tool, particularly in cases where the true coordinates of both the protein and ligand are unknown. Unlike traditional docking methods that require predefined structural input, AF3 operates based solely on 1-D composition information. This capability makes it especially useful for ligand docking scenarios where structural data is incomplete or unavailable. While further refinement could be needed to improve its accuracy, AF3 presents a great alternative for structure-free docking predictions.

#### 3.1.4 The Limitations in AF3′s Protein–PFAS Docking Performance are Primarily Driven by Inaccuracies in Ligand-Pocket Orientation and Ligand Structure Predictions

The primary sources of poor performance of AF3 ligand docking are incorrect ligand-pocket orientation and inaccurate ligand structure predictions, as shown in Figure S3. Among all poor docking attempts, errors in ligand-pocket orientation accounted for the largest proportion, comprising 58.6% in the “After Set” and 56.7% in the “Before Set”. This indicates that while AF3 can often predict the structures of both ligand and pocket in proximity with high accuracy, it struggles to correctly predict their binding orientation, leading to misaligned docking poses.

Following ligand-pocket orientation prediction, inaccurate ligand structure prediction represents the second most common contribution, accounting for 29.9% in the “After Set” and 28.4% in the “Before Set”, which is potentially due to limitations in handling ligand flexibility and diversity (Figure S3). Meanwhile, a poor pocket structure prediction are less frequent, occurring in 17.2% of cases in the “After Set” and 17.9% in the “Before Set”, indicating that AF3 is generally more reliable in modeling protein pocket structures than ligand conformations. When both ligand and pocket predictions exceed the RMSD threshold, the likelihood is much lower, accounting for only 5.7% of failures in the “After Set” and 3.0% in the “Before Set”. This suggests that while AF3 occasionally produces completely incorrect docking predictions, such instances are relatively low. Overall, these findings highlight that AF3′s primary limitations stem from the poor performance in binding orientation and ligand structure prediction, which should be a key area for future improvement to enhance its docking accuracy.

#### 3.1.5 Success Rate Can Be Significantly Increased by Selecting Multiple Top-Ranked Candidates Predicted by AF3 and Vina

To explore whether the success rate of pocket-aligned ligand pose prediction could be further improved, we evaluated the effect of selecting from multiple top-ranked candidates predicted by AF3 and hybrid strategies that incorporate Vina-based refinements (Figure 2). Selecting the minimal RMSD value of AF3′s top five predictions (“AF3-Top5”) led to a modest improvement over the best pose alone (“AF3-Best”), increasing the success rate from 74.5% to 81.0% in the “Before Set” and from 55.8% to 61.4% in the “After Set” (Figure 2). However, this improvement is statistically insignificant in the “After Set”, although significant in the “Before Set” (*p* < 0.05). Nevertheless, further enhancement can be achieved by integrating Vina and AF3. Notably, the hybrid strategies progressively improved success rates as the quality of the structural input increased, from Vina, Vina-1, to Vina-2. A common case is when the native protein structure is available; combining AF3-Top5 with Vina-1-Top5 yields an excellent performance, reaching 85.5% in the “Before Set” and 77.0% in the “After Set”, which is statistically significant (*p* < 0.001). These results demonstrate that selecting from multiple candidates can substantially improve docking accuracy, especially when high-quality individual structures are available. It is worth noting that using the “Top 5” poses may not be meaningful if some of them have significantly higher binding free energies in Vina. In such cases, applying an energy threshold may be a more effective strategy to determine which poses should be considered for further refinement.

**Figure 2:**
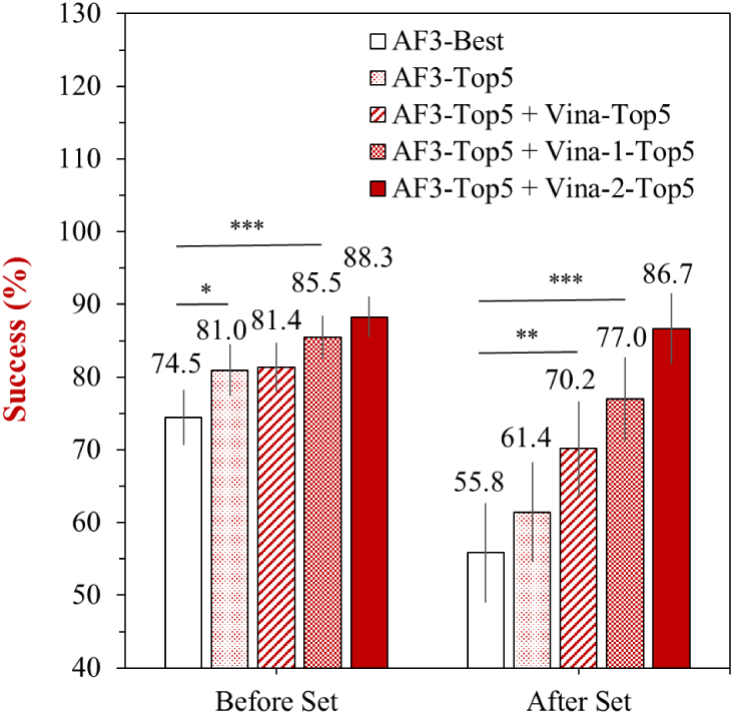
Improvement of success rates (%) through hybrid integration of AF3 and Vina predictions on the “Before Set” and “After Set”. “AF3-Best” refers to the top-ranked pose from AF3, and “AF3-Top5” represents the pose with the minimal RMSD value among the top-5 AF3 predictions. Hybrid strategies incorporate Vina’s top-5 predictions into AF3-Top5 selection: “AF3-Top5 + Vina-Top5” combines the top-5 predictions of Vina, “AF3-Top5 + Vina-1-Top5” combines Vina-1, and “AF3-Top5 + Vina-2-Top5” combines Vina-2. The average success rates are labeled above each bar, and error bars represent the 95% confidence intervals.

However, these docking results did not account for the potential impact of ligand flexibility, particularly in the case of flexible PFAS molecules. Molecular dynamics simulations could be a useful approach to further quantify and explore this flexibility. In addition, a more accurate approach is still needed to identify the most reliable pose. This can be achieved by integrating advanced models for binding free energy calculations, such as molecular mechanics Poisson–Boltzmann surface area (MM-PBSA) and generalized born surface area (MM-GBSA). Additionally, more precise free energy methods, such as free energy perturbation (FEP), can provide a rigorous assessment of protein–ligand binding stability and affinity, further improving the accuracy of interaction predictions.^19,35^ Future studies are needed to develop a more accurate and efficient combinational docking strategy for reliably identifying optimal protein–PFAS interactions.

### 3.2 Impact of PFAS Types on AF3 Docking Performance

Among all selected protein-PFAS systems, we further filtered out PFAS with distinct structural motifs and categorized them into three groups: (1) PFAS containing the −CF_2_–CF- motif, as defined by the US EPA,^34^ (2) PFAS with the -Ph-F motif, which features unique aromatic fluorine structures, and (3) PFAS with only −CF_2_- or −CF_3_ groups. Furthermore, we included additional systems containing environmentally relevant PFAS compounds and examined how common features, such as chain length and net charge, affect AF3′s docking performance.

#### 3.2.1 –CF_2_–CF– Type

The docking performance of AF3, compared to Vina, demonstrates relatively higher accuracy in evaluating the protein-PFAS systems containing the −CF_2_–CF– motif. As shown in Table 1, AF3 achieved low RMSD values (≤0.2 nm) in most cases when evaluating the protein backbone and pocket, reaffirming its strength in overall protein structure prediction. However, when ligand-specific metrics were considered, particularly pocket-aligned ligand RMSD, several entries showed poor performance (highlighted in bold), especially for the complex structures unseen in the training data set of the AF3 model (Table 1). Among the systems with only one ligand, AF3 performed poorly only on 4J03_FVS, which exhibited the highest RMSD values in both the “Ligand” and “Pocket_Aligned Ligand” metrics. This poor performance is likely due to the large size and high flexibility of the FVS ligand, but the binding pocket itself is accurately predicted by both AF3 and Vina (Figure S4). In contrast, other cases of poor AF3 performance were only observed in systems containing multiple ligands. Notably, these cases were specifically considered because they involve highly environmentally relevant PFAS compounds. To better understand why AF3 performed poorly in these cases, we collected seven protein–PFAS complexes and closely examined the best docking poses predicted by both AF3 and Vina. Three cases (4E99_P8S, 7AAI_8PF, and 7Z57_IGB), shown in Figure S5, involve the same human serum albumin (HSA) protein bound to different PFAS ligands. Another three cases (7FD7_4EI, 7FEK_8PF, and 7FEU_4I6), presented in Figures 3 and S6, feature the same heart-type fatty acid-binding protein (hFABP) bound to PFAS ligands with varying chain lengths. The last case (8U57_8PF) is a PPARγ–PFOA complex, where PPARγ refers to peroxisome proliferator-activated receptor gamma, and PFOA is perfluorooctanoic acid (Figure S7).

**Table 1:** Comparison of Docking Performance between AF3 and Vina for Protein–PFAS Interactions, with Each PFAS Containing a −CF_2_–CF– Motif^a^

		RMSD (nm)					
		AF3				Vina	
PDBID LigandID	Released Date	Protein Backbone	Protein Pocket	Ligand	Pocket_Aligned Ligand	Ligand	Pocket_Aligned Ligand
3RZ7_RZ7	8/10/2011	0.02	0.01	0.04	0.06	0.19	**0.68**
3RZ1_RZ1	8/10/2011	0.02	0.01	0.09	0.10	**0.21**	**0.59**
3RYZ_RYZ	8/10/2011	0.03	0.01	0.06	0.07	0.16	0.18
3RYX_RYX	8/10/2011	0.02	0.01	0.11	0.11	0.18	**0.41**
4E99_P8S*	6/6/2012	0.14	0.06	0.14	0.16	0.19	**0.21**
4J03_FVS	6/5/2013	0.08	0.05	**0.27**	**0.45**	**0.27**	**0.49**
5DDF_5A1	9/9/2015	0.11	0.02	0.12	0.13	0.17	**0.95**
6VQF_R7 V	4/8/2020	**0.35**	0.18	0.07	0.08	0.12	0.14
6RZX_KPQ	6/3/2020	0.02	0.01	0.02	0.03	0.03	**0.27**
7JTM_VK7	9/16/2020	**0.36**	0.23	0.09	0.11	0.09	0.11
7JYM_Z8I	11/25/2020	0.26	0.15	0.06	0.07	0.12	0.16
7AAI_8PF*	2/24/2021	**0.44**	0.08	0.14	**0.87** ^**#**^	0.17	**0.91**
7LUK_YDY	5/12/2021	**0.45**	**0.22**	0.09	0.13	0.12	0.17
7FD7_4EI*	7/20/2022	0.03	0.03	0.13	**0.48**	0.15	**0.43**
7FEK_8PF*	7/27/2022	0.03	0.02	0.17	**0.30**	0.18	**0.57**
7FEU_4I6	7/27/2022	0.03	0.02	0.12	0.13	0.15	**0.48**
7Z57_IGB*	10/12/2022	**0.46**	0.10	0.13	**0.22**	0.14	**0.28**
8U57_8PF*	7/24/2024	**0.54**	**0.22**	0.16	**0.80** ^**#**^	0.16	**0.83**

a The table shows PDB release dates and RMSD values (nm) based on four AF3 alignment references : protein backbone , protein pocket , ligand, and pocket aligned ligand . Vina results are shown for ligand and pocket-aligned ligand alignments. Bold values indicate poor predictions (RMSD > 0.2 nm). “*” denotes entries with multiple PFAS molecules, but one ligand was used in the docking process. “#” indicates that performance can be improved after docking multiple available ligands into the protein. The CSO residue was converted to CYS in 8U57_8PF.

**Figure 3:**
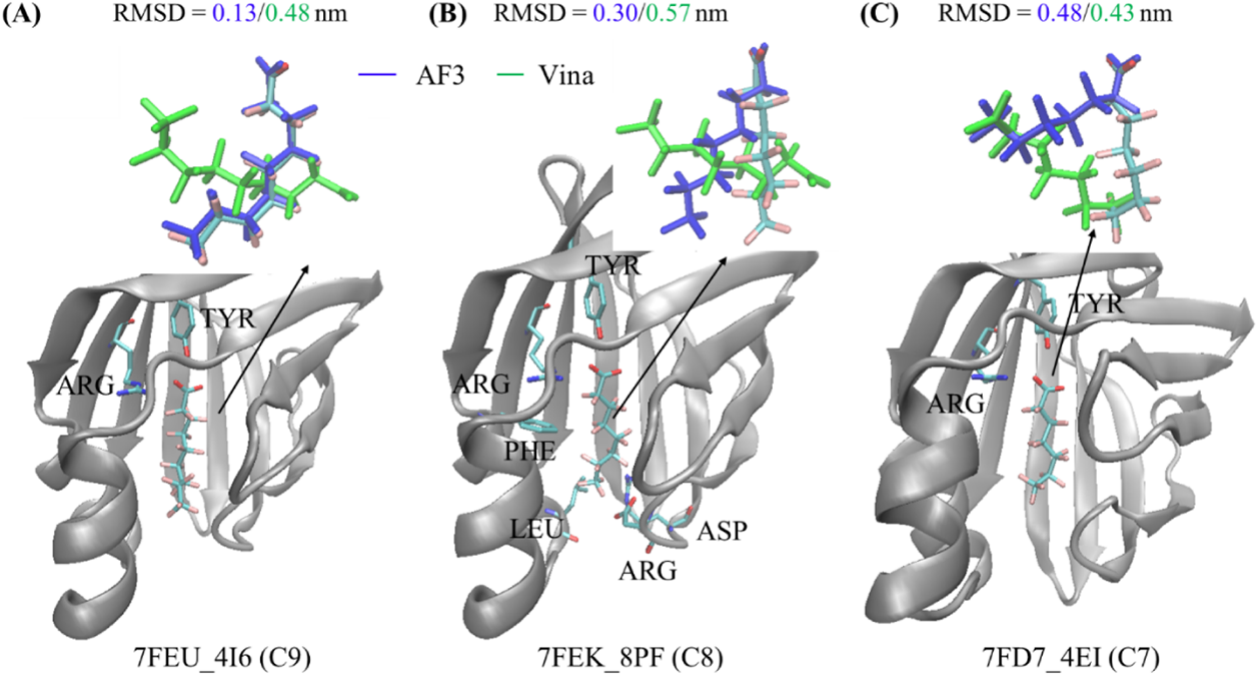
Structural comparison of representative protein–PFAS complexes predicted by AF3 and Vina. These examples feature the same heart-type fatty acid-binding protein bound to PFAS ligands of varying chain lengths. The corresponding PDB ID and PFAS ligand ID (4EI: C7, PFHpA; 8PF: C8, PFOA; and 4I6: C9, PFNA) are labeled at the bottom of each panel. Each panel shows the experimental native structure, with the protein rendered in NewCartoon and the ligand displayed in Licorice representation; the AF3-predicted ligand pose (blue), and the Vina-predicted pose (green). Key interacting residues within 0.3 nm of the ligand are labeled. RMSD values (in nm) between the predicted and experimental ligand poses are shown above each panel, respectively.

In the HSA–PFAS systems, for the two seen examples (4E99_P8S and 7AAI_8PF), both AF3 and Vina accurately predicted the ligand pose in 4E99_P8S, where PFOS contacts LYS, TYR, SER, LEU, PHE, and ASN within 0.3 nm of the binding site (Figure S5A). For 7AAI_8PF, AF3 correctly identified the binding pocket and placed the PFOA headgroup accurately, but the hydrophobic tail was misaligned, yielding a high RMSD (Figure S5B). This mismatch stems from docking a single ligand; when all ligands present in 7AAI_8PF were docked, the AF3 prediction closely matched the native structure, and the misalignment disappeared (Figure S5D), likely reflecting that 7AAI_8PF is seen in the training data. For the unseen example 7Z57_IGB, AF3 and Vina performed similarly, and the predicted structures were close to the native one (Figure S5C). We also tested whether including all ligands (two negatively charged IGB and four negatively charged MYR) in 7Z57_IGB could improve AF3 performance. It was observed that three MYR ligands matched their native sites, and one occupied the native site of an IGB ligand. However, both IGB ligands were poorly predicted, with one placed in a non-native binding site (Figure S5E).

In the hFABP–PFAS systems, AF3 achieved its best performance for 7FEU_4I6, as an unseen example, with the lowest RMSD among all systems, whereas Vina failed for this case (Figure 3A). AF3 successfully identified the protein binding pockets in all systems and consistently produced lower RMSD values than Vina. These RMSDs could be further reduced by selecting the most accurate pose from AF3′s top-5 predictions (Table S1). Additionally, AF3 consistently captured a key interaction between the PFAS headgroup and polar or positively charged residues, such as TYR and ARG. Interestingly, TYR–PFAS interactions were observed in all seven systems: in some cases, the headgroup interacted with TYR (e.g., 4E99_P8S, 7FEU_4I6, 7FEK_8PF, and 7FD7_4EI), while in others, the hydrophobic tail interacted with TYR (e.g., 7AAI_8PF, 7Z57_IGB, and 8U57_8PF). However, AF3 misaligned the hydrophobic tail of PFAS in both 7FEK_8PF and 7FD7_4EI, resulting in higher RMSD values (Figure 3B,C). To investigate this, we tested whether docking multiple available ligands could reduce the error. In the native structures, both systems contain two PFAS ligands. Docking both ligands partially restored the correct tail orientation for 7FEK_8PF but not for 7FD7_4EI (Figure S6B,C). AF3 also failed to reproduce a distinct binding mode observed for the second PFAS ligand in both systems. In the native structures, the fluorinated carbon chain (−C–F regions) of this ligand is buried within the protein, while the headgroup is exposed to the solvent. AF3 did not capture this arrangement; instead, it oriented the headgroup toward the binding pocket to interact with residues such as SER in 7FEK_8PF (Figure S6B) or ARG in 7FD7_4EI (Figure S6C). These results suggest that AF3 struggles to accurately predict such solvent-exposed headgroup interactions in unseen examples.

In the PPARγ–PFOA system as an unseen example, a previous study has reported that PFOA occupies three distinct binding sites: two within the PPARγ ligand-binding domain (LBD) and one within the activation function 2 (AF-2) region on the protein surface.^36^ The key native binding interactions within the PPARγ-LBD are shown in Figure S7D. The native structure exhibits strong intermolecular interactions between two PFOA molecules, including a very short O–F distance of ∼2.32 Å. None of the predictions successfully reproduced this binding interaction. For instance, docking a single PFOA ligand failed to predict the correct binding site in both AF3 and Vina (Figure S7A). Docking three PFOA ligands partially recovered the correct LBD site when using the CCD code of PFOA as input (Figure S7C), while using the negatively charged SMILES string of PFOA yielded slightly worse performance (Figure S7B). In both docking cases, AF3 failed to capture binding interactions within the AF-2 region or reproduce the native intermolecular contacts between two PFOA molecules. Interestingly, in the native structure of 8U57_8PF, the headgroup of another PFOA molecule is oriented toward the solvent, which was also not captured by AF3. Instead, in the AF3-predicted structures, the head groups of all three PFOA molecules preferentially interact with polar or positively charged residues, such as SER, GLN, HIS, and ARG (Figure S7E). This discrepancy may be due to the limited representation in AF3′s training data of PFAS ligands with solvent-exposed head groups.

To assess whether the protonation state of PFAS ligands can impact AF3 performance, we generated ligand inputs using SMILES strings to explicitly define their deprotonated (negatively charged) forms for these selected PFAS compounds (see Table S2). In most cases, the predicted binding poses were similar to those obtained using the original AF3 inputs. However, in certain cases, such as 7FEU_4I6 and 7Z57_IGB, the use of the deprotonated form led to reduced docking performance, although AF3-Top5 provided slightly better predictions (Table S3). We evaluated the impact of docking multiple ligands and found that, in most cases, using either the protonated or deprotonated form produced similar results, but exceptions were also observed. For instance, the deprotonated form improved predictions for 7FEK_8PF, whereas the protonated form improved predictions for 8U57_8PF. These findings suggest that AF3 may struggle to distinguish the potential effects of protonated versus deprotonated states of PFAS ligands, and it could often result in similar docking performance regardless of the protonation state. However, the current data set includes only 18 systems containing the −CF_2_–CF– motif, with 13 belonging to the training data and only 5 unseen data. This limited sample size makes it difficult to draw definitive conclusions about AF3′s reliability in handling this specific type of PFAS.

#### 3.2.2 –Ph–F Type

The docking accuracy of both AF3 and Vina remains consistently high for PFAS molecules containing the (−Ph–F) structural motif, with only two notable exceptions, as shown in Table S4. In the case of 6TND_8RH, the correct ligand pose can be also recovered by Vina-Top5, due to the input use of the AF3-predicted structure. It indicates that AF3 could effectively model PFAS compounds featuring aromatic fluorine substitutions. However, a significant outlier is observed in the 7G59_ZIS system, where all docking strategies, including AF3-Top5 and Vina-Top5, resulted in exceptionally high RMSD values (Table S5). This poor performance may be attributed to the large size and high conformational flexibility of the ZIS ligand. However, while AF3 and Vina generally perform well for PFAS molecules with the (−Ph–F) motif, additional data and validation are needed to confirm their robustness across a broader range of aromatic PFAS.

#### 3.2.3 Chain Length of PFAS

To assess the impact of PFAS chain length on AF3′s docking performance, we first examined three representative protein–PFAS complexes, all involving the same heart-type fatty acid-binding protein but differing in PFAS chain lengths (Figure 3). Across these systems, Vina consistently failed to capture the correct binding interactions as the PFAS chain length increased from C7 to C9. In contrast, AF3 showed more reliable performance, accurately predicting binding interactions between the PFAS headgroup and residues such as TYR and ARG. These interactions were consistently observed across all systems, including shorter-chain PFAS (C2–C6) and even the ultrashort-chain trifluoroacetic acid (Figure S6A). We also examined five other systems from the “Before Set” (3RZ7_RZ7, 3RZ1_RZ1, 3RYZ_RYZ, 3RYX_RYX, and 6RZX_KPQ), all involving PFAS compounds of varying chain lengths or head groups bound to human carbonic anhydrase II.^37,38^ In these cases, the neutral PFAS headgroup consistently formed specific interactions with a THR residue (Figure S8), regardless of chain length (Figure S8A–D) or headgroup (Figure S8B,E). Under these conditions, AF3 showed strong and consistent docking performance, likely because they were included in its training data.

#### 3.2.4 Net Charge of PFAS

To evaluate whether the net charge of PFAS ligands influences AF3′s docking performance, we recalculated success rates based on ligand charge: negative, neutral, and positive (Figure S9). In the “Before Set,” AF3 showed relatively high success across all charge types, with best pose success rates of 76.5% for negatively charged PFAS, 70.4% for neutral PFAS, and 80.4% for positively charged PFAS. However, these differences were not statistically significant when compared to the overall performance across all ligands (74.5%). A comparable decline in success rate was observed in the “After Set,” with values decreasing to 48.0% for negative, 58.5% for neutral, and 53.7% for positive PFAS compounds. Again, no statistically significant differences were detected among these groups. These results suggest that the net charge of PFAS could have an insignificant impact on AF3′s predictive performance.

### 3.3 AF3 Computing Performance

We also present the correlation between protein size, measured in the number of residues, and the elapsed computation time for AF3 docking predictions in the “Before Set” and “After Set” (Figure S10). A general trend of increasing computation time with protein size is observed, though some variations exist, particularly for proteins within the midsize range. AF3 demonstrates the ability to handle protein systems with fewer than 1000 residues within approximately 1000 s, making it a computationally efficient tool for protein–ligand docking studies. This suggests that AF3 can be used for routine docking tasks, providing a practical balance between computational cost and accuracy. Even for larger protein systems, AF3 maintains reasonable execution times, highlighting its scalability and feasibility for medium-to-large protein docking applications.

Interestingly, the relationship between protein size and computation time is not strictly linear. Several proteins within the 200–400 residue range exhibit higher-than-expected computation, sometimes exceeding those of larger proteins. This suggests that additional factors, such as sequence composition, may contribute to variations in computational demands. Despite these inconsistencies, AF3 remains a feasible tool for virtual screening in a high-throughput manner. Given the high-throughput capability of traditional Vina docking, integrating AF3 with Vina could provide an accurate and efficient combinational strategy for refining the docking space in protein-PFAS systems.

## Supplementary Material



## Notes

**Figures S1–S10** present the workflow, performance, and structural comparisons for AF3 and Vina in predicting protein–PFAS interactions. They include analyses of chain length, ligand charge, binding site variations, and computation time across data sets. **Tables S1–S5** compare AF3 and Vina docking performance for protein–PFAS interactions, evaluating the effects of ligand structural motifs (−CF_2_–CF–, −Ph–F) and protonation states across protein-PFAS complexes (PDF)
