# Supplementary material for: Assessing AlphaFold 3 for Per- and Polyfluoroalkyl Substances Docking in Protein Structures
Source: Environ Sci Technol. 2025 Aug 26;59(35):18440–9. doi: 10.1021/acs.est.5c03917 (PMC12424161; doi:10.1021/acs.est.5c03917)
Supplement: Supplementary file 1 [file es5c03917_si_001.pdf]

Supporting Information

Assessing AlphaFold 3 for Per- and Polyfluoroalkyl Substances Docking in Protein Structures

Xiping Gong [1], Hualu Zhou [2], Qingguo Huang [1,*]

1. Department of Crop and Soil Sciences, College of Agricultural and Environmental Sciences, University of Georgia, Griffin, Georgia 30223, United States

2. Department of Food Science and Technology, College of Agricultural and Environmental Sciences, University of Georgia, Griffin, Georgia 30223, United States

*__Corresponding author__: Qingguo Huang

Email address: qhuang@uga.edu

ASSOCIATED CONTENT

Supporting Information: It has 17 pages, 10 figures, and 5 tables. **Figures S1–S10** present the workflow, performance, and structural comparisons for AF3 and Vina in predicting protein–PFAS interactions. They include analyses of chain length, ligand charge, binding site variations, and computation time across datasets. **Tables S1–S5** compare AF3 and Vina docking performance for protein–PFAS interactions, evaluating effects of ligand structural motifs (–CF_2–CF–, –Ph–F) and protonation states across protein-PFAS complexes.

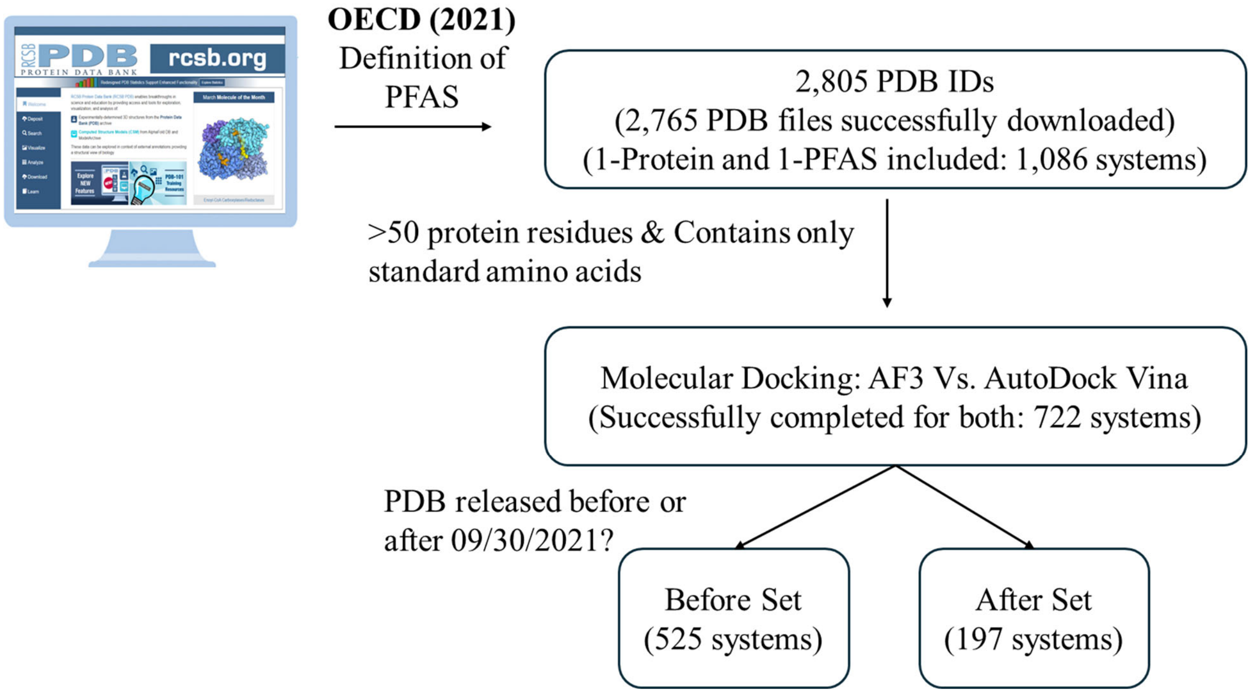

Figure S1. Workflow for protein-PFAS system preparation and docking analysis. Protein structures are retrieved from the PDB and undergo preprocessing, including structure refinement and ligand extraction. The two datasets, "Before Set" and "After Set," were categorized based on release date, protein size, inclusion of only standard amino acids, and successfully processed by both AF3 and Vina.

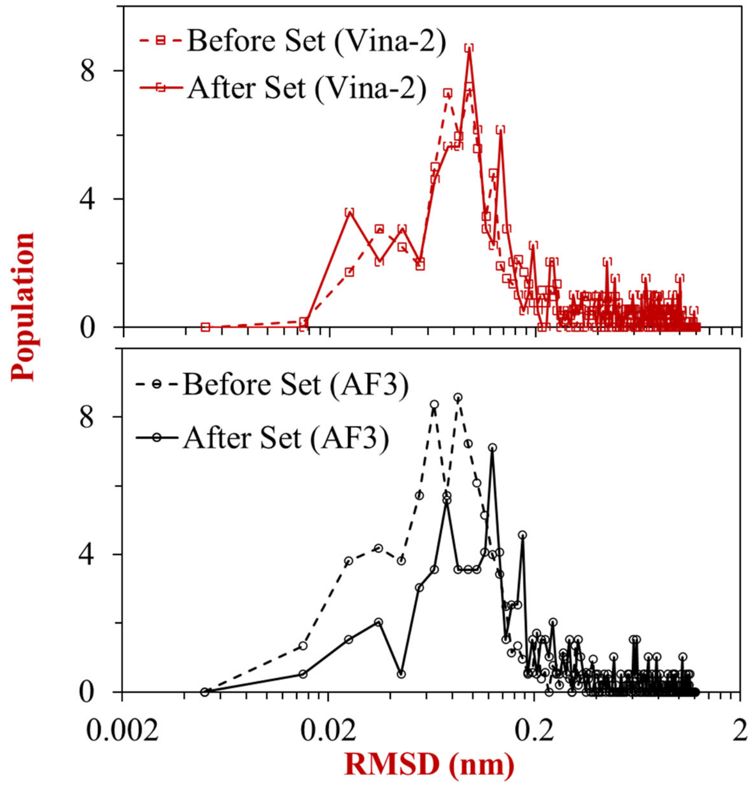

Figure S2. Distribution of heavy-atom RMSD values for pocket-aligned ligands predicted by AF3 and Vina-2 on the “Before Set” and “After Set.” The population represents the frequency of samples within each RMSD range. The top panel shows the population distribution for Vina-2 predictions, while the bottom panel shows the distribution for AF3 predictions. The “Before Set” and “After Set” are indicated by dashed and solid lines, respectively. The distributions highlight differences in docking accuracy across datasets and methods.

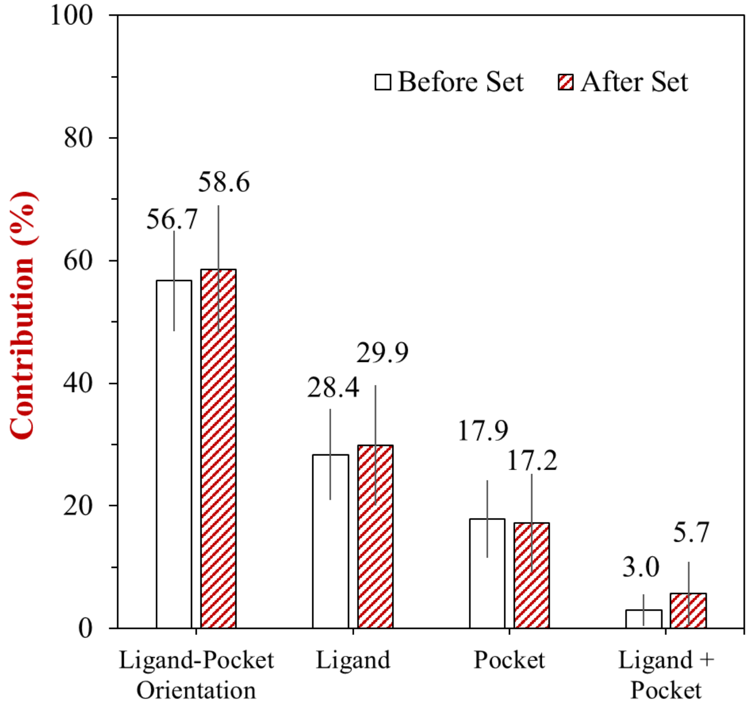

Figure S3. Contribution of four distinct components to the poor performance of AF3 in predicting protein–PFAS interactions, where the RMSD of the pocket-aligned ligand exceeds 0.2 nm. The components include: "Ligand-Pocket Orientation," where both the aligned protein pocket and ligand have RMSD ≤ 0.2 nm, but the pocket-aligned ligand exceeds 0.2 nm; "Ligand," where the aligned ligand has RMSD > 0.2 nm; "Pocket," where the aligned protein pocket has RMSD > 0.2 nm; and "Ligand + Pocket," where both the aligned protein pocket and ligand have RMSD > 0.2 nm. Average percentages are labeled above each bar, with error bars representing the 95% confidence intervals.

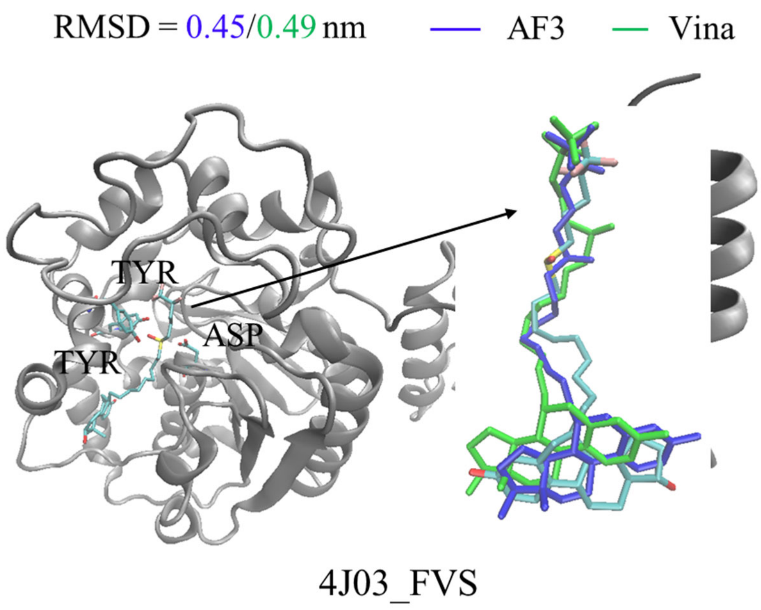

Figure S4. Structural comparison of 4J03_FVS complex predicted by AF3 and Vina. The corresponding PDB ID and ligand ID are labeled at the bottom of each panel. Each panel shows the experimental native structure (gray), with the protein rendered in NewCartoon and the ligand displayed in Licorice representation, the AF3-predicted ligand pose (blue), and the Vina-predicted pose (green). Key interacting residues are labeled, including those with heavy atoms within 0.3 nm of the ligand's heavy atoms. RMSD values (in nm) between the predicted and experimental ligand poses are shown above each panel, respectively.

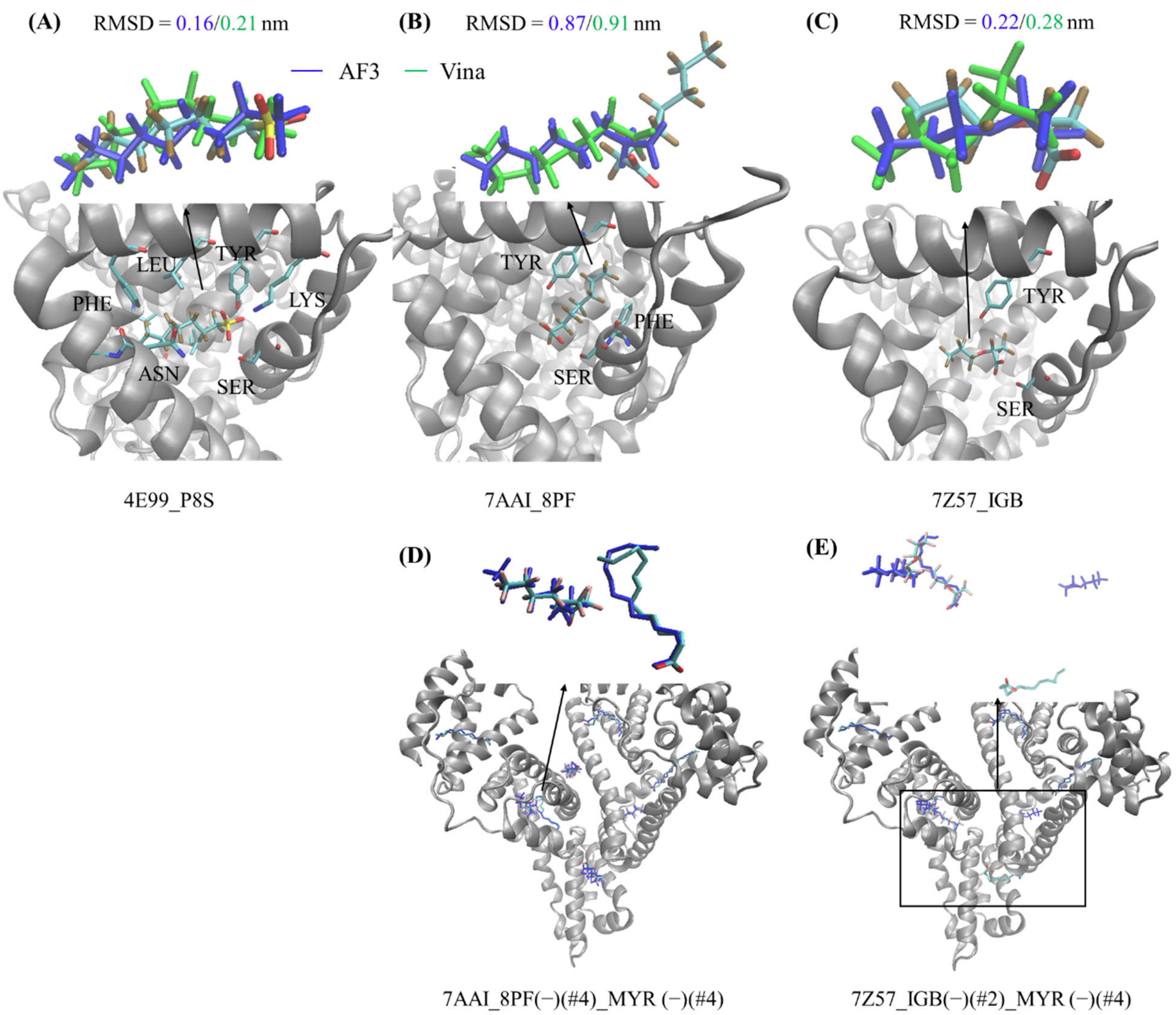

Figure S5. Structural comparison of representative human serum albumin (HSA) protein–PFAS complexes predicted by AF3 and Vina. (**A-C**) The interactions between the HSA protein with three PFAS ligands (P8S: PFOS; 8PF: PFOA; IGB: GenX). (**D**) Predicted structures after docking four negatively charged 8PF ligands and four negatively charged MYR ligands. (**E**) Predicted structures after docking two negatively charged IGB ligands and four negatively charged MYR ligands. The corresponding PDB ID and ligand ID are labeled at the bottom of each panel. Each panel shows the experimental native structure (gray), with the protein rendered in NewCartoon and the ligand displayed in Licorice representation, the AF3-predicted ligand pose (blue), and the Vina-predicted pose (green). Key interacting residues are labeled, including those with heavy atoms within 0.3 nm of the ligand's heavy atoms. RMSD values (in nm) between the predicted and experimental ligand poses are shown above each panel, respectively.

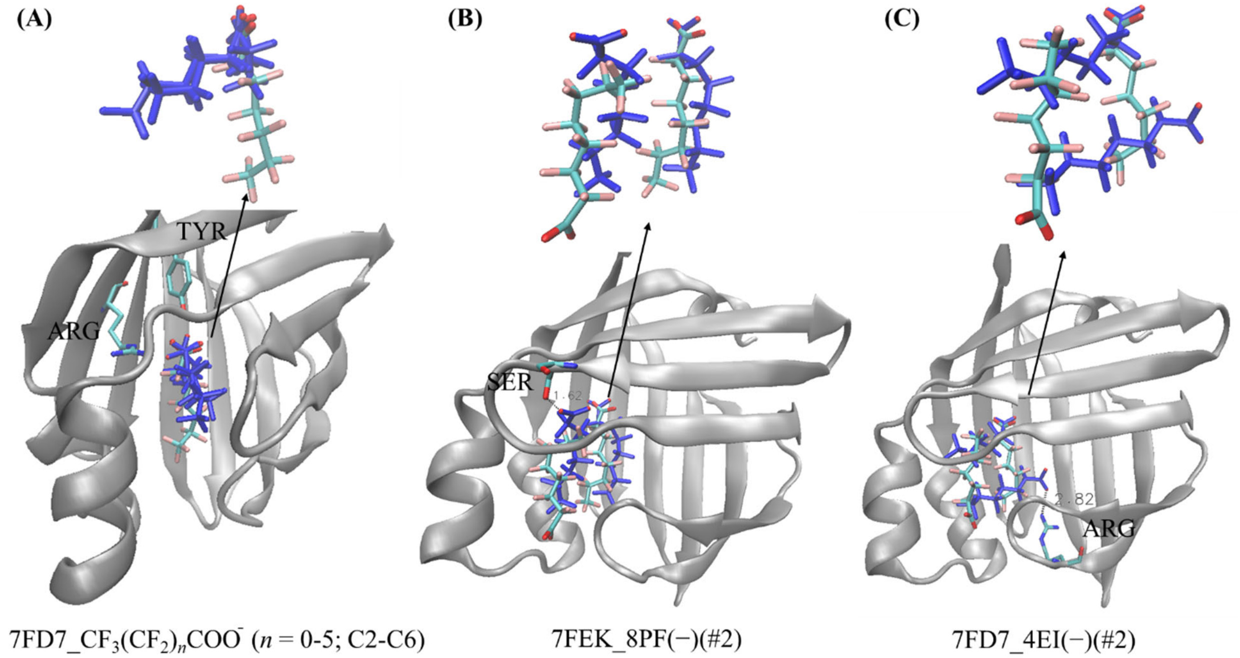

Figure S6. (**A**) AF3 performance in predicting the binding of heart-type fatty acid-binding protein to negatively charged PFAS ligands of varying chain lengths (C2-C6). One PFAS structure was used for each AF3 docking. (**B-C**) AF3 performance in predicting the binding of heart-type fatty acid-binding protein to two negatively charged 8PF ligands (**B**) and two negatively charged 4EI ligands (**C**). The corresponding PDB ID and PFAS ligand ID are labeled at the bottom. Each panel shows the experimental native PDB structure, with the protein rendered in NewCartoon and the ligand displayed in Licorice representation, and the AF3-predicted ligand poses (blue). Key interacting residues within 0.3 nm of ligand are labeled.

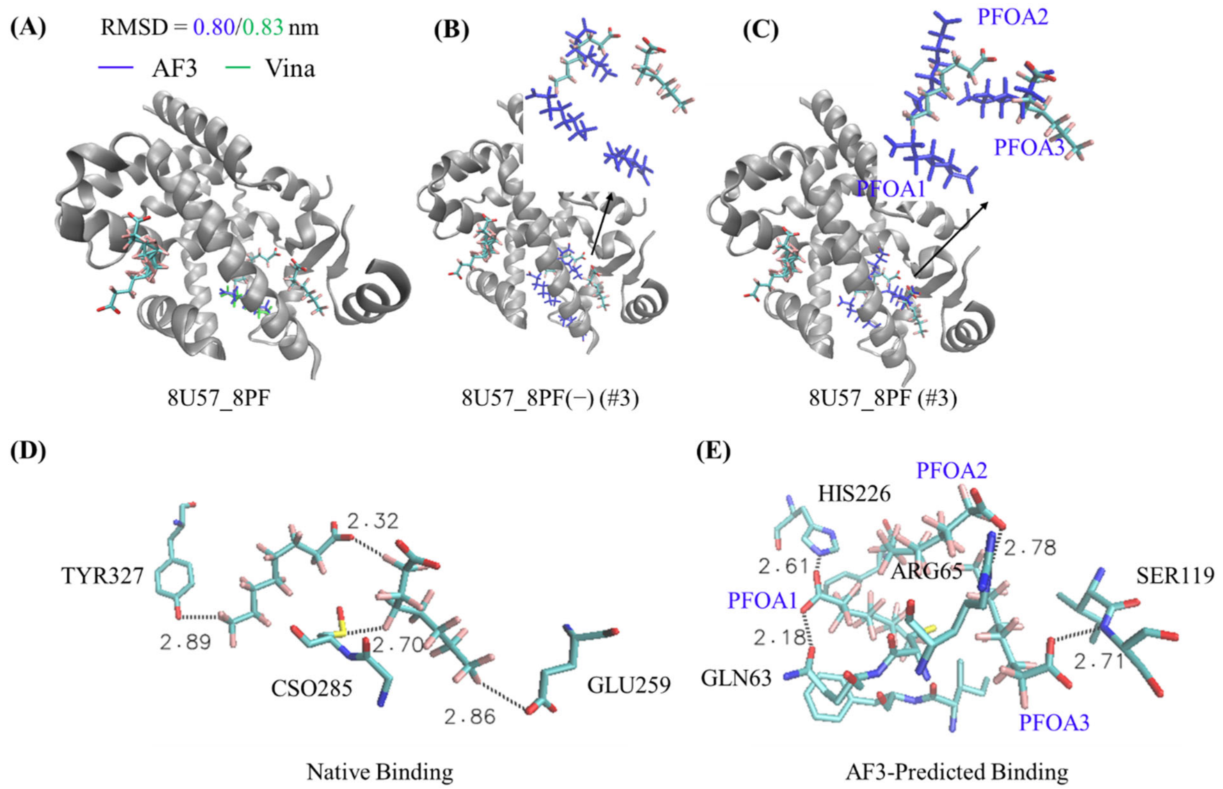

Figure S7. (**A**) Structural comparison of the PPARγ–PFOA complex predicted by AF3 and Vina. PPARγ refers to peroxisome proliferator-activated receptor gamma, and PFOA to perfluorooctanoic acid. (**B**) AF3 prediction of the PPARγ–PFOA(−) (#3) complex using the SMILES string of negatively charged PFOA, where three ligands were used for docking. (**C**) AF3 prediction of the PPARγ–PFOA (#3) complex using the CCD code of PFOA. (**A–C**) Each panel displays the experimental native structure in gray (protein in NewCartoon and ligand in Licorice representation) alongside the AF3-predicted ligand pose in blue. CSO residue was converted to CYS for AF3 docking. (**D**) Key interactions between two PFOA molecules and protein residues in the native PPARγ–PFOA system. (**E**) Key interactions between three PFOA molecules and protein residues in the AF3-predicted 8U57_8PF (#3) system.

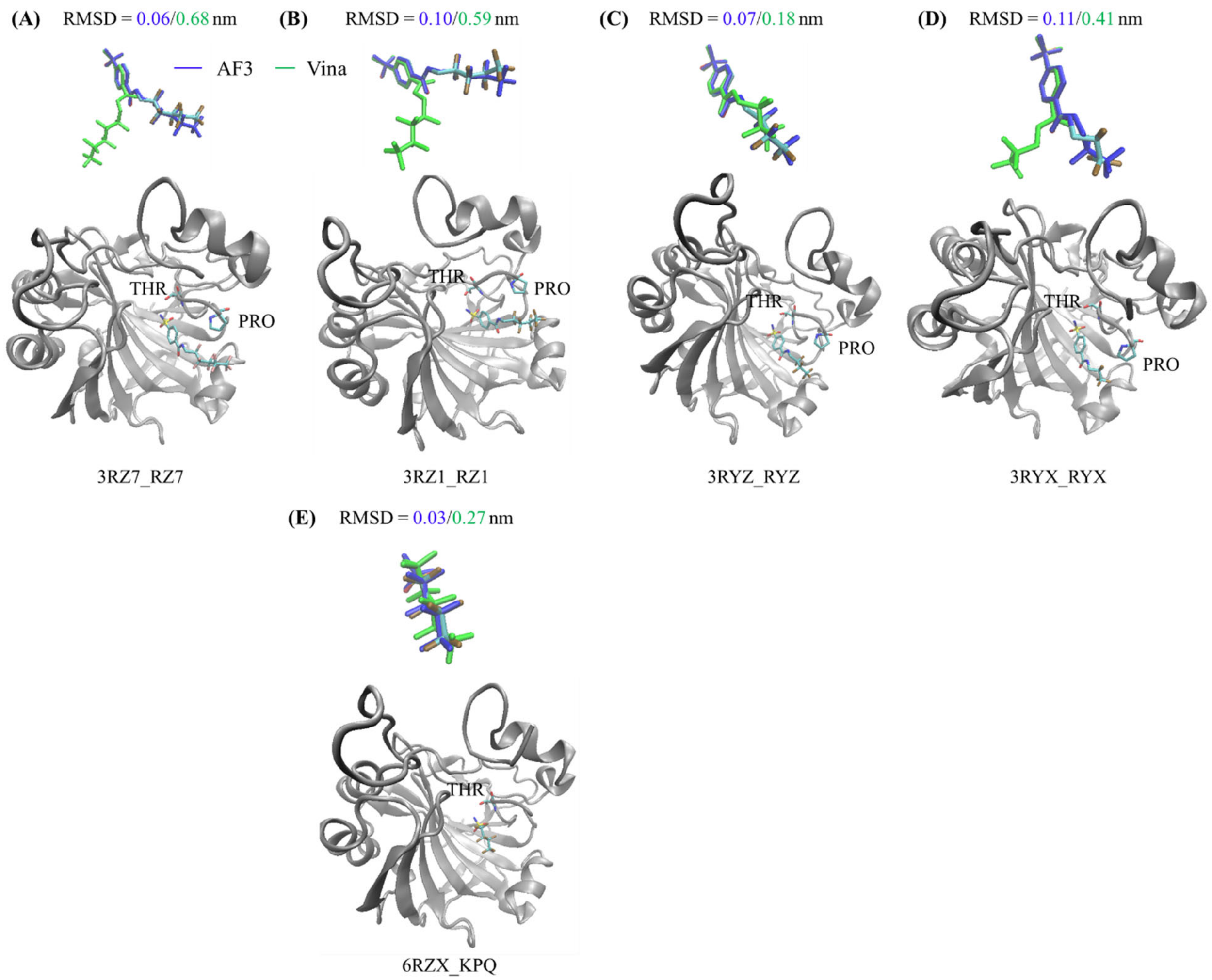

Figure S8. Structural comparison of representative protein–PFAS complexes predicted by AF3 and Vina. These examples feature the same human carbonic anhydrase II protein (HCA II) bound to PFAS ligands of varying chain lengths or head groups (RZ7: $CF_3(CF_2)_4C_8H_9N_2O_3S$; RZ1: $CF_3(CF_2)_3C_8H_9N_2O_3S$; RYZ: $CF_3(CF_2)_2C_8H_9N_2O_3S$; RYX: $CF_3(CF_2)_1C_8H_9N_2O_3S$; and KPQ: $CF_3(CF_2)_3SO_2NH_2$). The corresponding PDB ID and PFAS ligand ID are labeled at the bottom of each panel. Each panel shows the experimental native structure (gray), with the protein rendered in NewCartoon and the ligand displayed in Licorice representation, the AF3-predicted ligand pose (blue), and the Vina-predicted pose (green). Key interacting residues within 0.3 nm of ligand heavy atoms are labeled. RMSD values (in nm) between the predicted and experimental ligand poses are shown above each panel, respectively.

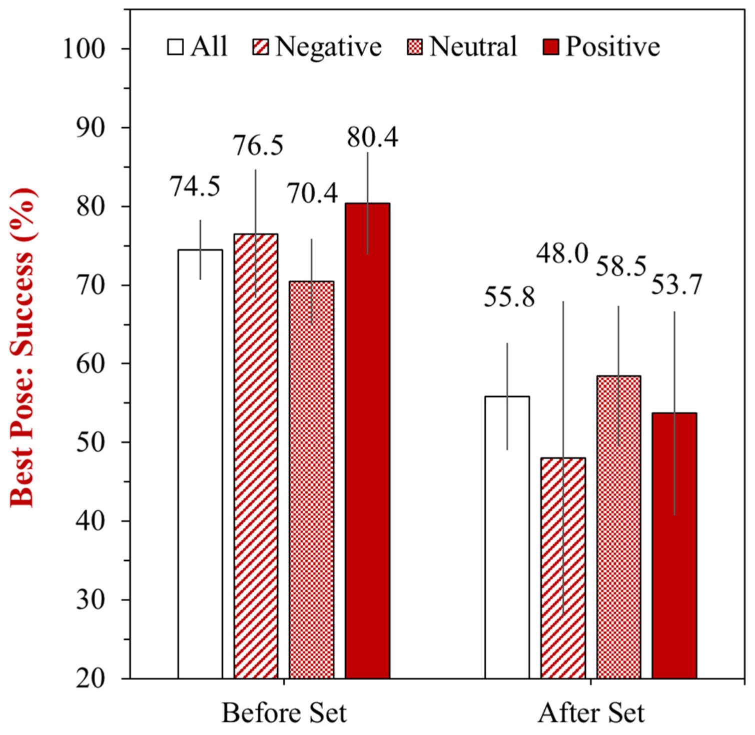

Figure S9. Success rates (%) of AF3 on the "Before Set" and "After Set," categorized by the net charge of PFAS ligands. The net charge of each PFAS molecule was determined using Open Babel by assigning protonation states at pH 7.4. Results are shown for all ligands combined (dataset size: 525 for "Before Set" and 197 for "After Set"), and separately for negatively charged (75/12), neutral (193/69), and positively charged (123/29) PFAS compounds, where values represent the number of samples in the "Before Set" and "After Set", respectively. Average success rates are labeled above each bar, and error bars represent the 95% confidence intervals.

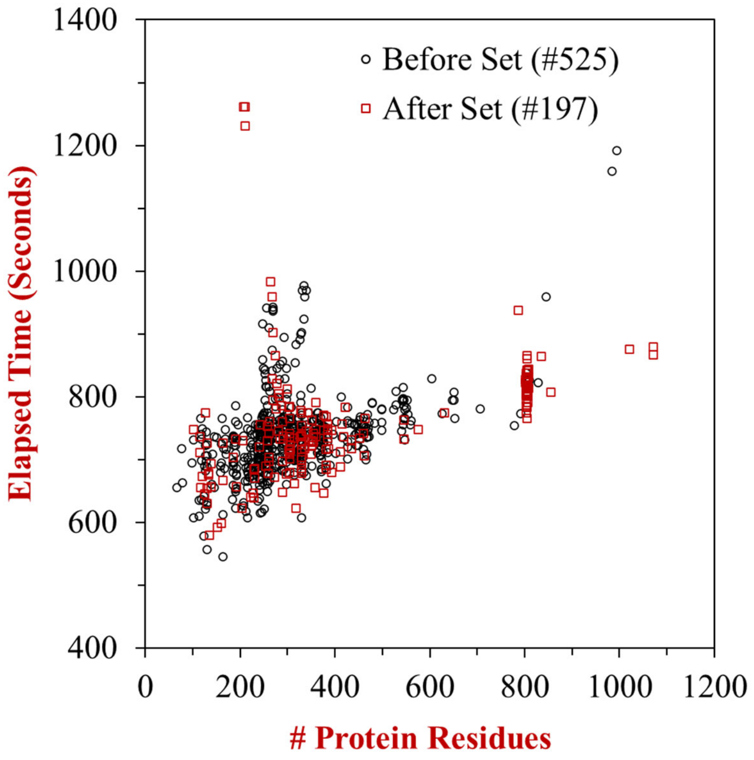

Figure S10. Correlation between the number of protein residues and elapsed computation time (second) for AF3 docking in the “Before Set” and “After Set”. Each point represents an individual docking case. The elapsed time reflects the full execution duration for AF3-based docking predictions. Four models were generated using a predefined seed, with computations performed on 32 CPUs and a single A100 GPU.

Table S1. Comparison of docking performance between AF3-Top5 and Vina-Top5 in predicting protein–PFAS interactions, where each PFAS molecule contains a characteristic $–CF_2–CF–$ structural motif. The table lists the release date of each PDB entry and the RMSD values (in nm) for four AF3-based structural alignment references: protein backbone, protein pocket, ligand, and pocket-aligned ligand. Corresponding RMSD values from Vina predictions are reported for ligand and pocket-aligned ligand alignments. Values in bold indicate poor predictions where the RMSD exceeds 0.2 nm. “*” denotes entries with multiple PFAS molecules, but one ligand was used in the docking process. “#” indicates that performance can be improved after docking multiple available ligands into the protein. CSO residue was converted to CYS in 8U57_8PF.

		RMSD (nm)					
		AF3-Top5				Vina-Top5	
**PDBID_LigandID**	**Released Date**	**Protein Backbone**	**Protein Pocket**	**Ligand**	**Pocket_Aligned Ligand**	**Ligand**	**Pocket_Aligned Ligand**
3RZ7_RZ7	8/10/2011	0.02	0.01	0.02	0.03	0.14	**0.57**
3RZ1_RZ1	8/10/2011	0.02	0.01	0.02	0.03	0.16	**0.45**
3RYZ_RYZ	8/10/2011	0.03	0.01	0.07	0.07	0.15	0.17
3RYX_RYX	8/10/2011	0.02	0.01	0.03	0.04	0.11	0.13
4E99_P8S*	6/6/2012	0.14	0.05	0.13	0.14	0.16	0.18
4J03_FVS	6/5/2013	0.07	0.05	0.07	0.15	**0.27**	**0.49**
5DDF_5A1	9/9/2015	0.06	0.02	0.11	0.13	0.12	**0.52**
6VQF_R7V	4/8/2020	**0.31**	0.17	0.07	0.08	0.12	0.14
6RZX_KPQ	6/3/2020	0.02	0.01	0.02	0.02	0.03	0.05
7JTM_VK7	9/16/2020	0.11	0.08	0.09	0.11	0.09	0.11

7JYM_Z8I	11/25/2020	0.16	0.11	0.04	0.07	0.12	0.16
7AAI_8PF*	2/24/2021	**0.43**	0.08	0.14	**0.85**#	0.15	**0.87**
7LUK_YDY	5/12/2021	0.22	0.12	0.09	0.13	0.12	0.17
7FD7_4EI*	7/20/2022	0.03	0.02	0.09	**0.45**	0.11	**0.42**
7FEK_8PF*	7/27/2022	0.03	0.02	0.16	**0.23**	0.17	**0.52**
7FEU_4I6*	7/27/2022	0.02	0.02	0.09	0.10	0.13	0.14
7Z57_IGB*	10/12/2022	**0.44**	0.10	0.13	0.19	0.14	**0.25**
8U57_8PF*	7/24/2024	**0.34**	0.17	0.16	**0.77**#	0.16	**0.76**

Table S2. Comparison of docking performance of AF3 in terms of different protonated states in predicting protein–PFAS interactions across 7 complexes. For the “AF3” calculations, the ligands were in their protonated state, as indicated by the CCD description of each ligand ID. In contrast, for the “AF3 (ligand charged)” calculations, we used SMILES strings to ensure that the ligands were in their negatively charged (deprotonated) form. The table lists the release date of each PDB entry and the RMSD values (in nm) for four AF3-based structural alignment references: protein backbone, protein pocket, ligand, and pocket-aligned ligand. Corresponding RMSD values from Vina predictions are reported for ligand and pocket-aligned ligand alignments. Values in bold indicate poor predictions where the RMSD exceeds 0.2 nm. “*” denotes entries with multiple PFAS molecules, but one ligand was used in the docking process. “#” indicates that performance can be improved after docking multiple available ligands into the protein. CSO residue was converted to CYS in 8U57_8PF.

		**RMSD (nm)**							
		**AF3 (protonated)**				**AF3 (deprotonated)**			
**PDBID_LigandID**	**Released Date**	**Protein Backbone**	**Protein Pocket**	**Ligand**	**Pocket_Aligned Ligand**	**Protein Backbone**	**Protein Pocket**	**Ligand**	**Pocket_Aligned Ligand**
4E99_P8S[*]	6/6/2012	0.14	0.06	0.14	0.16	0.14	0.05	0.17	0.19
7AAI_8PF[*]	2/24/2021	**0.44**	0.08	0.14	**0.87**[#]	**0.45**	0.08	0.16	**0.86**[#]
7FD7_4EI[*]	7/20/2022	0.03	0.03	0.13	**0.48**	0.03	0.02	0.19	**0.48**
7FEU_4I6	7/27/2022	0.03	0.02	0.12	0.13	0.03	0.02	0.20	**0.24**
7FEK_8PF[*]	7/27/2022	0.03	0.02	0.17	**0.30**	0.03	0.02	0.18	**0.30**[#]
7Z57_IGB[*]	10/12/2022	**0.46**	0.10	0.13	**0.22**	**0.44**	0.10	0.17	**2.79**
8U57_8PF[*]	7/24/2024	**0.54**	**0.22**	0.16	**0.80**[#]	**0.35**	0.17	0.16	**0.81**

Table S3. Comparison of docking performance of AF3-Top5 in terms of different protonated states in predicting protein–PFAS interactions across 7 complexes. For the “AF3-Top5” calculations, the ligands were in their protonated state, as indicated by the CCD description of each ligand ID. In contrast, for the “AF3-Top5 (deprotonated)” calculations, we used SMILES strings to ensure that the ligands were in their negatively charged (deprotonated) form. The table lists the release date of each PDB entry and the RMSD values (in nm) for four AF3-based structural alignment references: protein backbone, protein pocket, ligand, and pocket-aligned ligand. Corresponding RMSD values from Vina predictions are reported for ligand and pocket-aligned ligand alignments. Values in bold indicate poor predictions where the RMSD exceeds 0.2 nm. “*” denotes entries with multiple PFAS molecules, but one ligand was used in the docking process. “#” indicates that performance can be improved after docking multiple available ligands into the protein. CSO residue was converted to CYS in 8U57_8PF.

		**RMSD (nm)**							
		**AF3-Top5 (protonated)**				**AF3-Top-5 (deprotonated)**			
**PDBID_LigandID**	**Released Date**	**Protein Backbone**	**Protein Pocket**	**Ligand**	**Pocket_Aligned Ligand**	**Protein Backbone**	**Protein Pocket**	**Ligand**	**Pocket_Aligned Ligand**
4E99_P8S*	6/6/2012	0.14	0.05	0.13	0.14	0.13	0.05	0.13	0.15
7AAI_8PF*	2/24/2021	**0.43**	0.08	0.14	**0.85**#	**0.43**	0.08	0.16	**0.86**#
7FD7_4EI*	7/20/2022	0.03	0.02	0.09	**0.45**	0.03	0.02	0.19	**0.47**
7FEU_4I6	7/27/2022	0.02	0.02	0.09	0.10	0.02	0.02	0.19	**0.23**
7FEK_8PF*	7/27/2022	0.03	0.02	0.16	**0.23**	0.03	0.02	0.13	**0.30**#
7Z57_IGB*	10/12/2022	**0.44**	0.10	0.13	0.19	**0.44**	0.10	0.17	**2.71**
8U57_8PF*	7/24/2024	**0.34**	0.17	0.16	**0.77**#	**0.35**	0.17	0.16	**0.22**

Table S4. Comparison of docking performance between AF3 and Vina in predicting protein–PFAS interactions across 7 complexes, where each PFAS molecule contains a characteristic –Ph–F structural motif. The table lists the release date of each PDB entry and the RMSD values (in nm) for four AF3-based structural alignment references: protein backbone, protein pocket, ligand, and pocket-aligned ligand. Corresponding RMSD values from Vina predictions are reported for ligand and pocket-aligned ligand alignments. Values in bold indicate poor predictions where the RMSD exceeds 0.2 nm.

		**RMSD (nm)**					
		**AF3**				**Vina**	
**PDBID_LigandID**	**Released Date**	**Protein Backbone**	**Protein Pocket**	**Ligand**	**Pocket_Aligned Ligand**	**Ligand**	**Pocket_Aligned Ligand**
3B67_B67	9/9/2008	0.02	0.02	0.20	0.20	0.20	0.20
6TND_8RH	5/13/2020	0.11	0.16	0.08	0.10	0.19	**0.88**
3VHE_42Q	11/2/2011	0.09	0.03	0.01	0.03	0.06	0.08
3B5R_B5R	9/9/2008	0.03	0.02	0.07	0.07	0.07	0.08
4D89_BXD	11/21/2012	0.07	0.06	0.06	0.06	0.08	0.09
7NP5_UKB	6/2/2021	0.05	0.06	0.13	0.13	0.14	0.15
7G59_ZIS	12/18/2024	0.13	0.03	0.20	**1.57**	0.17	**1.59**

Table S5. Comparison of docking performance between AF3-Top5 and Vina-Top5 in predicting protein–PFAS interactions across 7 complexes, where each PFAS molecule contains a characteristic –Ph–F structural motif. The table lists the release date of each PDB entry and the RMSD values (in nm) for four AF3-based structural alignment references: protein backbone, protein pocket, ligand, and pocket-aligned ligand. Corresponding RMSD values from Vina predictions are reported for ligand and pocket-aligned ligand alignments. Values in bold indicate poor predictions where the RMSD exceeds 0.2 nm.

		**RMSD (nm)**					
		**AF3-Top5**				**Vina-Top5**	
**PDBID_LigandID**	**Released Date**	**Protein Backbone**	**Protein Pocket**	**Ligand**	**Pocket_Aligned Ligand**	**Ligand**	**Pocket_Aligned Ligand**
3B67_B67	9/9/2008	0.03	0.02	0.04	0.05	0.07	0.09
6TND_8RH	5/13/2020	0.11	0.16	0.08	0.10	0.08	0.12
3VHE_42Q	11/2/2011	0.09	0.03	0.01	0.03	0.06	0.08
3B5R_B5R	9/9/2008	0.03	0.02	0.07	0.07	0.07	0.08
4D89_BXD	11/21/2012	0.07	0.02	0.03	0.03	0.08	0.09
7NP5_UKB	6/2/2021	0.04	0.04	0.01	0.04	0.14	0.15
7G59_ZIS	12/18/2024	0.12	0.04	0.16	**1.57**	0.15	**1.46**
